# Amongst patients taking biologic therapies for axial spondyloarthritis, which factors are associated with work non-participation?

**DOI:** 10.1186/s12891-020-03247-9

**Published:** 2020-04-06

**Authors:** Tom Nadin, Dinny Wallis, Christopher R. Holroyd, Stefania D’Angelo, Karen Walker-Bone, Christopher J. Edwards

**Affiliations:** 1 Sheffield Teaching Hospitals NHS Foundation Trust, Sheffield, UK; 2 University Hospital Southampton NHS Foundation Trust, Southampton, UK; 3 MRC Versus Arthritis Centre for Musculoskeletal Health and Work, MRC Lifecourse Epidemiology Unit, University Hospital Southampton NHS Foundation Trust, Southampton, UK; 4 NIHR Clinical Research Facility, University Hospital Southampton NHS Foundation Trust, Southampton, UK

**Keywords:** Axial spondyloarthritis, Work participation, Biologic therapies, Disease activity, Productivity, Absenteeism

## Abstract

**Background:**

Axial spondyloarthritis (axSpA) frequently presents during working age and therefore impacts work participation. Biologic therapies have demonstrated a positive impact on work-related outcomes in clinical trials but real world data are limited. Therefore, we investigated the prevalence and predictors of work impairment and disability among axSpA patients attending a biologic therapy clinic.

**Methods:**

This was a single-centre, cross-sectional study of patients with axSpA treated with biologic therapy. Work participation was assessed with the Work Productivity and Activity Impairment (WPAI) Questionnaire. Work outcomes (presenteeism, absenteeism, health-related job loss) were compared for gender, time since diagnosis, smoking status and disease outcome measures.

**Results:**

Data were available for 165 patients (mean age 47.6 years, 75% male, 21% current smokers). Mean time since diagnosis was 15.5 years and mean duration of biologic therapy 4.7 years; 19/165 (11.5%) were on a tapered-dose regimen. Occupational data were available for 144 patients amongst whom 101 (70.1%) were either currently employed or in full time education. Of those eligible to work, 17/118 (14.4%) reported inability to work due to their axSpA. Amongst those in employment, 10.8% reported absenteeism due to axSpA in the week prior to their clinic visit (mean hours missed = 13). The mean work productivity impairment was 23%. Higher disease activity (BASDAI) and markers of global health, quality of life and pain, (BAS-G, ASQoL and spinal pain VAS) were associated with axSpA related job loss, absenteeism and presenteeism.

**Conclusions:**

In this group of axSpA patients on biologic therapy (mean age 47.6 years), almost 1 in 6 (14.4%) reported axSpA related job loss. Poor work outcomes: axSpA-related work disability, absenteeism and presenteeism were associated with poorer scores for patient-reported disease outcome measures. Strategies for enhancing work productivity should be directed towards those patients at risk of poor work outcomes. More data are needed including details of the types of work that are most difficult with axSpA.

## Background

Work is a key determinant of self-esteem, social identity and standing within the community quite apart from its impact on financial circumstances [1]. Worklessness is associated with debt, suicide, self-harm and poorer prospects for the children of those who are affected. Axial spondyloarthritis (axSpA), which frequently presents when patients are in their second or third decade of life causes back pain and disability and is recognised to impact work participation [2, 3].

To date, much of the research in this field has come from analysis of secondary work outcomes amongst patients participating in pharmaceutically-funded randomised controlled trials [4–10]. In this setting, absenteeism has been the most commonly evaluated outcome measure. A recent UK prospective cohort study identified that starting biologic therapy was associated with improvement in work outcomes compared to those not starting such therapy, but the negative impact on work was still substantial [11]. A few researchers have recently reported work participation in patients with early axSpA [12, 13] and these have suggested some impact of the disease on work participation but do not provide any information about the impact of longstanding disease or whether outcomes can be improved by early, effective disease control. Therefore, we investigated the rates of work participation, productivity and absenteeism, as well as the risk factors for these poor work outcomes, amongst a group of patients with established spondyloarthritis on treatment with biologic therapies.

## Methods

Patients were eligible for this cross-sectional study if they were at least 18 years old and fulfilled the Assessment of SpondyloArthritis International Society (ASAS) criteria for axSpA [14]. All were under rheumatological follow-up twice annually and they were all currently being treated for axSpA using biologic therapies (adalimumab, certolizumab, etanercept, golimumab, infliximab).

Between July and December 2016, participants attending the Southampton Biological Therapies Review Service provided information about their employment status, smoking status and completed the following patient-reported outcome measures: the Bath Ankylosing Spondylitis Disease Activity Index (BASDAI), Bath Ankylosing Spondylitis Functional Index (BASFI), Bath Ankylosing Spondylitis Global Score (BAS-G) and Ankylosing Spondylitis Quality of Life Questionnaire (ASQoL). Back pain was assessed using a 10 cm visual analogue scale. Bath Ankylosing Spondylitis Metrology Index (BASMI) was recorded in the clinic.

Absenteeism and productivity were assessed using the Work Productivity Activity Index:Specific Health Problem Questionnaire (WPAI) [15]. This questionnaire has been validated to assess the impact of inflammatory rheumatic diseases including ankylosing spondylitis on productivity at work [16]. It consists of a 6-item questionnaire which allows calculation of absenteeism, presenteeism and work productivity loss. Presenteeism allows the assessment of the proportion of lost productivity in the workplace attributable to the disease (Q5), rated on a scale 0–10 (reported as a % productivity). Absenteeism (Q2) is defined as the number of hours ‘off sick’ from the job attributable to the disease (and not due to other reasons) and is calculated from the formula: number of hours missed at work due to axSpA /total number of hours that they should have been scheduled to work during the past 7 days.

### Data analysis

We performed analyses of 3 outcomes: “absenteeism” was defined as the percentage of work time missed due to health and categorised as 0 vs at least some time; “presenteeism” was defined as the percentage of impairment while working and was also dichotomised (≤40% vs > 40%). Finally, axSpA-related job loss was defined as not working due to the disease vs being in work/student. Participants’ characteristics were described according to the outcomes and differences between groups were tested with chi-squared tests, Mann-Whitney U test, or t test depending on the nature of the variable.

The associations between disease outcome measures and work outcomes were analysed using logistic regression with results expressed as odds ratios (ORs) and 95% confidence intervals (95%Cis). After initial univariate analyses (not shown in the tables) estimates were adjusted for age, gender, smoking status and number of different biologic therapies used. Data were analysed using Stata 14.2.

## Results

In total, data were available for 165 axSpA patients (123 men and 42 women). Their mean age was 47.6 years (standard deviation 13.1 years). On average, they had been diagnosed with axSpA for 15.5 years (standard deviation 12.1 years). Amongst these, 69 men and 15 women reported that they had ever smoked cigarettes and 29 men and 6 women were current smokers. All were taking biologic therapies with a mean duration of 4.7 years (standard deviation 3.3 years). The median scores for each of the outcomes were: BASDAI 3.7, BASFI 3.5, BASMI 3.4, BAS-G 3.0, ASQoL 5.0 and back pain VAS 4.0 cm.

**Fig. 1:**
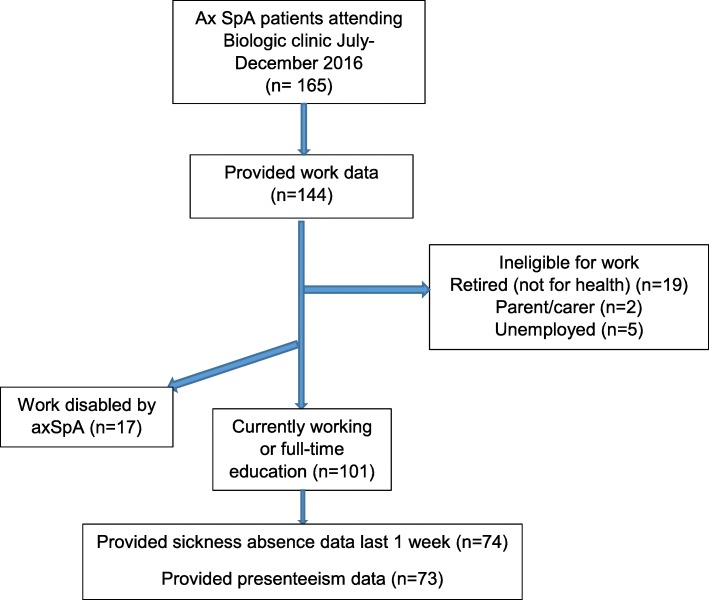
Analysis of work participation among a cohort of 165 patients with axSpA on biologics

In total, 144 people provided occupational information (Fig. 1): 5 patients were unemployed, 2 were full-time carers and 19 were retired for non-health reasons. Of the remainder, 99 patients reported that they were currently in paid work (77 full-time and 22 part-time) and 2 that they were currently in full-time education and 17 (14.4%) reported that they were work disabled because of axSpA.

### Absenteeism and presenteeism amongst current workers

Amongst those who were current workers or in full time education (*n* = 101) and who provided data (*n* = 74), sickness absence due to axSpA was reported by 8 people (10%) with a mean of 13 h of work lost during that week. In univariate models, absenteeism in the past week was associated with younger age (36.9 years vs. 45.3 years, OR = 0.9, 95%CI = 0.8 to 0.9); poorer scores for ASQoL (OR associated to 1 unit increase in the score = 1.3, 95% CI = 1.1 to 1.5) and poorer scores for BASFI (OR associated with 1 unit increase in the score = 1.4, 95% CI = 1.0 to 2.0) but no other factors (not gender, smoking or other disease outcome measures) (Table 1). Also, the 73/101 workers who provided presenteeism data reported an average productivity loss of 23% in the past week. When comparison was made of those with low rates of productivity loss (≤40%) vs. those with higher rates (> 40%), the univariate analyses showed that younger age (39.1 years vs. 45.8 years, OR 0.9, (95% CI 0.9–1.0)), being a current smoker (80.0% vs. 19.7%, OR 16.3, (95% CI 3.1–87.0)), having a shorter time between diagnosis of axSpA and treatment (0.8 years vs. 6.8 years, OR 0.8, (95% CI 0.7–1.0)), being prescribed 2 biologic therapies vs 1 for axSpA (50.0% vs. 14.8%, OR 7.2, (95% CI1.3–38.3)), higher disease activity (BASDAI 5.4 vs 2.6, OR 2.5, (95% CI 1.6–4.1)); and worse quality of life (ASQoL 12.0 vs. 4.0, OR 1.5, (95% CI 1.2–1.8)) were associated with productivity loss (Table 1). There was no significant difference in BASDAI, BASFI, BASMI, BAS-G or ASQoL between patients who provided data about work and those who did not.

**Table 1:** Factors associated with absenteeism, presenteeism and axSpA-related job loss in a population of patients receiving biologics

Factors	Current workers (***n*** = 101)								Currently eligible to work (***n*** = 118)			
	Absenteeism (Completed by ***n*** = 74)				Presenteeism (Completed by ***n*** = 73)							
	None reported (*n* = 66) (%)	Absent in past week (*n* = 8) (%)	*p*-value	crude OR (95%CI)	≤40% (*n* = 62) (%)	> 40% (*n* = 11) (%)	*p*-value	crude OR (95%CI)	Currently working (*n* = 101)(%)	axSpA related job loss (*n* = 17)(%)	*p*-value	crude OR (95%CI)
Age (years)	45.3 (1.3)	36.9 (3.3)	0.03		45.8 (1.4)	39.1 (2.7)	0.04		45.0 (1.1)	48.3 (2.2)	0.26	
Males, N(%)	49 (74.2)	5 (62.5)	0.48		47 (73.4)	9 (69.2)	0.76		76 (75.3)	13 (76.5)	0.91	
Current smoker, N(%)	17 (26.2)	4 (57.1)	0.09		12 (19.7)	8 (80.0)	< 0.001		26 (26.5)	5 (29.4)	0.81	
Time between diagnosis and treatment, Median (IQR)	5.6 (0.9,14.6)	1.9 (0.7,4.6)	0.21		6.8 (1.2,17.5)	0.8 (0.05,1.9)	0.004		5.6 (1.3,15.4)	4.5 (0.8,13.7)	0.79	
N treatment^a^												
1	45 (79.0)	2 (40.0)	0.14		43 (79.6)	3 (37.5)	0.04		60 (76.0)	9 (52.9)	0.003	
2	9 (15.8)	2 (40.0)			8 (14.8)	4 (50.0)			14 (17.7)	2 (11.8)		
3 or 4	3 (5.3)	1 (20.0)			3 (5.6)	1 (12.5)			5 (6.3)	6 (35.3)		
BASDAI, Median (IQR)	3.0 (1.6,4.7)	3.9 (2.4,7.0)	0.15	1.4 (0.9,2.0)	2.6 (1.6,4.1)	5.4 (4.7,7.4)	< 0.001	2.5 (1.6,4.1)	3 (1.6,5.1)	7.2 (5.9,8.0)	< 0.001	2.3 (1.6,3.5)
BASFI, Median (IQR)	2.4 (1.5,4.1)	4.2 (2.6,7.7)	0.08	1.4 (1.0,2.0)	2.1 (1.2,3.3)	5.3 (4.5,7.3)	< 0.001	2.0 (1.4,2.9)	2.8 (1.7,4.5)	7.4 9 (6.7,9.0)	< 0.001	2.6 (1.6,4.2)
BASMI, Median (IQR)	3.2 (1.6,4.6)	2.6^b^	0.83	0.8 (0.2,3.4)	3.0 (1.6,4.0)	2.6 (1.0,4.2)	0.69	0.8 (0.4,2.0)	2.8 (1.6,4.0)	6.0 (5.4,6.6)	0.01	9.3 (0.8112.7)
BASG, Median (IQR)	3.0 (1.0,4.0)	4.0 (1.0,7.0)	0.41	1.2 (0.9,1.7)	2.0 (1.0,3.0)	6.0 (4.5,8.5)	< 0.001	2.0 (1.4,2.9)	3.0 (1.0,5.0)	7.0 (6.0,9.0)	< 0.001	2.2 (1.5,3.1)
ASQoL, Median (IQR)	4.0 (1.0,8.0)	10.0 (4.0,16.0)	0.01	1.3 (1.1,1.5)	4.0 (0,7.0)	12.0 (9.30,17.0)	< 0.001	1.5 (1.2,1.8)	4.0 (2.0,8.0)	15.0 (12.5,16.0)	< 0.001	1.4 (1.2,1.7)

^a^Data for number of treatments were not available for 12 patients (absenteeism analysis); 11 patients (presenteeism analysis). ^b^Only one patient with sickness absence had BASMI recorded

### Work disability caused by axSpA

In total, 17 patients reported that they were not working because of their axSpA (rate of health-related premature work loss = 14.4%). A comparison of those working or in full-time education (*n* = 101) with those who had stopped work because of axSpA showed that the work-disabled tended to be slightly older (mean age 48.3 years vs. 45.0 years, *p* = 0.26) and were more likely to have been prescribed 3 or 4 different biologic therapies rather than 1. Moreover, all of the disease outcome measures were associated so that those who were work-disabled had higher BASDAI, BASMI, BASFI, BAS-G and ASQoL scores (Table 1). The relationships between work loss and disease outcome measures were further explored in multivariable analyses, adjusting for age, gender, smoking and number of prescribed drugs (Table 2). After adjustment, higher scores for BASDAI, BASFI, BAS-G and ASQoL were all significantly associated with having stopped working due to axSpA. No significant association was seen for BASMI and work disability.

**Table 2:** Adjusted multivariable analyses of the associations between disease outcome measures and axSpA-related job loss

Outcome measure	OR^a^	95% CI	***p***-value
BASDAI	3.2	1.7–6.1	< 0.001
BASFI	3.1	1.6–6.0	0.001
BAS-G	2.7	1.5–4.6	< 0.001
ASQoL	1.8	1.2–2.6	0.002

^a^Analyses adjusted for age, gender, smoking status and number of drug treatments

## Discussion

In this group of axSpA patients receiving biologic therapies, we found significant levels of work disability with almost 1 in 6 (14.4%) of patients (mean age 47.6 years) having stopped work because of their condition. AxSpA related job loss was significantly associated with higher scores for disease outcome measures, including BASDAI, BASFI, BAS-G and ASQoL, after adjustment for age, gender, smoking status and number of biologic therapies prescribed. Amongst those in work, 10% reported recent absenteeism (mean 13 h in past week) and the mean productivity loss was 23% in the past week. Both these outcomes were associated with poorer scores for disease activity, function, global health and quality of life. Taken together, these results suggest that older patients with more resistant disease requiring multiple biologics and poorer patient-reported disease outcome measures suffer more disability for work and are at increased risk of premature work loss.

Strengths of our study include the large number of patients with axSpA providing work data in a real-world setting, and the use of validated work-related outcome measures. However, there are several limitations. At the time of the patient visits, not all patients had radiographs available for review. We are therefore not able to describe the radiographic phenotype at the time of the visit. Extra-articular manifestations were not described in this study. A comparator group not taking biologic therapies was not included and the cross-sectional methodology means that temporal relationships between work outcomes and disease outcomes, and the effect of biologic therapies on work outcomes, could not be investigated. Missing data may have affected the results: although responders and non-responders for patient-reported work outcome measures did not differ with regard to disease outcome measures, there may be other unmeasured differences. A single-centre study such as this may not be generalisable to the entire axSpA population.

The ability to stay in work has been identified as a priority by people with axSpA [17]. Patients are aware of the impact of disease on work: in a survey of 770 Italian patients with spondyloarthritis (including axSpA), [18] 21% of patients reported having to change or leave their job, or losing their job, because of SpA. Discrimination was reported by 14% of patients and 14% believed that their disease had an impact on their salary. Loss of work productivity is an important component of the indirect costs of axSpA. The cost of productivity losses over the first 3 years of follow up were evaluated in an early SpA cohort [19]. While the most significant cost component in the study overall was biologic drugs, the costs of work productivity loss represented between 10 and 17% of annual costs and were highest in year 1 when 30% of patients incurred productivity losses, as compared with 24% in year 3.

Identification of those patients at high risk of poor work outcomes may allow healthcare teams to develop strategies to enhance work participation. The factors which predict adverse work outcomes have been investigated in a number of axSpA cohorts. In an Italian cohort of patients with early disease [13], work productivity loss was associated with increased disease activity and loss of functional ability. Disease activity was correlated with absenteeism, presenteeism and work productivity loss. The authors noted that absenteeism was lower than that reported by patients in other countries (7.9%, compared with 28% in a Dutch cohort [19]). In an observational study of patients starting etanercept for AS [20], fatigue was found to independently predict presenteeism and activity impairment but not work productivity loss or absenteeism.

In an analysis of 1188 patients who were working at the time of recruitment to a British axSpA cohort (BSRBR-AS) [21], the authors investigated whether presenteeism was associated with future absenteeism, and whether absenteeism was a risk factor for later leaving the workforce. The data supported both hypotheses. Several factors were associated with absenteeism: prior presenteeism, a labour-intensive job and peripheral joint involvement. Prior absenteeism was the only significant factor associated with leaving work 12 months later.

A cross-sectional study of patients in the Netherlands with chronic low back pain [12] found that the impact of disease on work outcomes in early, undiagnosed axSpA was not significantly different from that in patients with chronic low back pain. In both groups, pain and functional limitation were associated with work impairment. In these patients with untreated axSpA, it might be expected that work outcomes would improve with biologic therapy. The question of whether biologic therapy improves work outcomes in axSpA has been investigated in a number of RCTs which have demonstrated improvements in workplace and household productivity. However, patients recruited to RCTs may not be representative of the real-world axSpA population treated with biologic therapies. Observational data have only recently started to emerge. In the BSRBR-AS cohort [11], propensity matching was used to compare patients starting biologic therapy with those not starting such therapy. The authors found that patients undergoing biologic therapy experienced significantly greater improvements in presenteeism, work impairment and activity impairment than the untreated patients. However, at 12 months follow up, the impact of disease on work was still greater in the biologic treated cohort than in patients not treated with biologic therapies. The investigators performed a meta-analysis including their own study and 4 other studies in which patients had been treated with biologic therapies compared to placebo or non-biologic treatments and which reported work outcomes in (total of 1109 patients) [22]. They found that presenteeism, work impairment and activity impairment outcomes improved more in patients receiving biologic therapies than in those not receiving such therapies. There was little improvement in absenteeism and no significant difference in the change in absenteeism between the groups. Biologic therapy did not result in a significant improvement in work instability (measured in 2 studies).

## Conclusions

While there is some evidence that treatment with biologic therapy can improve work outcomes, in this group of axSpA patients on biologic therapy there remains a significant loss in work productivity which is associated with patient-reported disease outcome measures.. Strategies for enhancing work productivity should be directed towards those patients at risk of job loss, absenteeism and presenteeism. More data are needed about the types of work which are most difficult with axSpA.

## Data Availability

The datasets analysed may be available from the corresponding author on reasonable request.

## Abbreviations

axSpA: axial spondyloarthritis
WPAI: Work Productivity and Activity Impairment;
BASDAI: Bath Ankylosing Spondylitis Disease Activity Index
BASFI: Bath Ankylosing Spondylitis Functional Index
BASMI: Bath Ankylosing Spondylitis Metrology Index
BAS-G: Bath Ankylosing Spondylitis Global Score
ASQoL: Ankylosing Spondylitis Quality of Life
VAS: Visual Analogue Scale
ASAS: Assessment of Spondyloarthritis International Society
